# Downregulation of cancer stem cell properties via mTOR signaling pathway inhibition by rapamycin in nasopharyngeal carcinoma

**DOI:** 10.3892/ijo.2015.3100

**Published:** 2015-07-21

**Authors:** CHUNGUANG YANG, YUE ZHANG, YU ZHANG, ZIHENG ZHANG, JIANHUA PENG, ZHI LI, LIANG HAN, QUANJIE YOU, XIAOYU CHEN, XINGWANG RAO, YI ZHU, ZHISU LIAO

**Affiliations:** 1 Department of Otolaryngology, Head and Neck Surgery, Xiang Ya Hospital, Central South University, Changsha, Hunan, P.R. China; 2 Department of Otolaryngology, The First Affiliated Hospital of Wenzhou Medical University, Wenzhou, Zhejiang, P.R. China; 3 Department of Otolaryngology, Dong Feng Hospital, Hubei University of Medicine, Shiyan, Hubei, P.R. China

**Keywords:** rapamycin, CD44, cancer stem cells, nasopharyngeal carcinoma, mTOR signaling pathway

## Abstract

Rapamycin, a mammalian target of rapamycin (mTOR) signaling inhibitor, inhibits cancer cell proliferation and tumor formation, including in nasopharyngeal carcinoma (NPC), which we proved in a previous study. However, whether rapamycin affects cancer stem cells (CSCs) is unclear. In examining samples of NPCs, we found regions of CD44-positive cancer cells co-expressing the stem cell biomarker OCT4, suggesting the presence of CSCs. Following this, we used double-label immunohistochemistry to identify whether the mTOR signaling pathway was activated in CD44-positive CSCs in NPCs. We used a CCK-8 assay and western blotting to explore whether the stem cell biomarkers CD44 and SOX2 and the invasion protein MMP-2 could be suppressed by treatment with rapamycin in cultured primary NPC cells and secondary tumors in BALB/c nude mice. Interestingly, we found that rapamycin inhibited mTOR signaling in addition to simultaneously downregulating the expression of CD44, SOX2 and MMP-2 and that it affected cell growth and tumor size and weight both *in vitro* and *in vivo*. Collectively, we confirmed for the first time that CSC properties are reduced and invasion potential is restrained in response to mTOR signaling inhibition in NPC. This evidence indicates that the targeted inhibition of CSC properties may provide a novel strategy to treat cancer.

## Introduction

Nasopharyngeal carcinoma is a type of epithelial carcinoma that is located in the nasopharynx and can easily invade the skull base and metastasize. A high incidence of NPC exists mainly in Southern China and Southeast Asia (~15–30 cases per 100,000 persons per year), but NPC is rare in Western and North American countries. There are many factors associated with the incidence of NPC, such as ethnic genetic differences, Epstein-Barr virus, and the consumption of salted fish and nitrosamine-containing foods (1–3). With newer strategies for treating NPC, the 3-year survival rate of NPC patients who are sensitive to radiation therapy is high (there are various survival rates corresponding to different regions, ranging from 48 to 83.5%). Some cases of advanced-stage cancer will be lethal because of skull base and neck lymph node invasion and distant metastasis (1,4,5). Therefore, some subpopulation of cancer cells may not be affected by radio- or chemotherapy against NPC; these cells are called CSCs (6).

Within an overall population of cancer cells, the CSC subpopulation, which is capable of strengthening proliferation, invasion, metastasis, tumorigenesis, heterogeneity and therapeutic resistance in tumors (7, 8), is very small. These cells also possess the ability to initiate tumor growth, and they maintain tumor self-renewal, even when they are implanted into mice after many passages, such as in the cases of leukemia (9) and brain cancer (10). However, the origin of CSCs is still unclear. Some evidence suggests that they arise from adult stem cells because they possess biomarkers that are similar to such cells (11); other evidence shows that CSCs are subpopulations of progenitor cells (12). It has also been argued that they originate from dedifferentiated cancer cells (13,14). However, opinions regarding their origin have not yet reached a consensus. Additionally, researchers are seeking new biomarkers to label and track CSCs. For instance, CD133 is a biomarker that was first confirmed in acute leukemia. CD133-positive cancer cells have stem cell properties, including indefinite growth, self-renewal and maintenance of tumor mass (15). CD44 is a CSC biomarker that has been identified in head and neck squamous cell carcinoma (HNSCC), in which CD44-positive cells possess the CSC properties of self-renewal and differentiation (16). In NPC, CD44 has also been confirmed as a biomarker of CSCs. CD44-positive cells have a higher survival rate than that of CD44-negative cells after treatment with cisplatin and docetaxel (17). The embryonic stem cell markers SOX2 and OCT4 also play substantial roles in the regulation of CSCs in ovarian carcinoma (18) and HNSCC (19), respectively.

mTOR is a serine/threonine kinase that is included in the PI3K/AKT/mTOR cascade; it is activated by phosphorylation. mTOR signaling regulates the downstream transcription factors ribosomal protein p70, S6 kinase and eIF4E-binding protein 1 (4E-BP1) and maintains cell growth and proliferation (20,21). Many studies have demonstrated that the mTOR signaling pathway plays an important role in regulating CSC expression and survival (22,23).

In this study, we investigated CD44 expression in NPC cells and determined that CD44-positive cells also expressed OCT4 in human tumor tissue samples. Then, we examined whether mTOR signaling was activated in NPC cells *in vitro* and *in vivo*. Furthermore, we used rapamycin to block mTOR signaling to inhibit the proliferation and tumorigenesis of NPC cells and to assess its potential to inhibit CD44, SOX2 and OCT4 in cultured primary NPC cells and secondary tumors. Additionally, we examined whether the expression of the invasion proteins MMP-2 and MMP-9 was suppressed by blocking mTOR signaling in secondary NPC tumors and whether tumor sizes and weights were also affected. Briefly, rapamycin affected various properties of NPC CSCs, which provides an indirect strategy for targeting CSCs to treat cancers.

## Materials and methods

### Nasopharyngeal tumor tissues

Human NPC specimens were obtained from the patients who underwent surgical resection at the Department of Otolaryngology, the First Affiliated Hospital, Wenzhou Medical University between 2005 and 2013. Tumors were diagnosed and classified at the Department of Pathology of the hospital by the pathologists according to the World Health Organization (WHO) guideline (24). Patients ranged in age from 31 to 81 years with a mean of 55.9 years. The tumors were obtained and fixed in 10% formaldehyde and embedded in paraffin for histopathological and immunohistochemical analysis. All studies on patients were conducted following the protocol approved by the Institutional Research Review Board at Wenzhou Medical University, China.

### Cell culture and drug test

The NPC cells were cultured from the primary tumor tissue isolated from a 54-year-old male patient, after obtaining written informed consent under protocols approved by the Human Research and Ethics Committee of the First Affiliated Hospital, Wenzhou Medical University. The NPC cells were cultured in the medium RPMI-1640 (Gibco, USA) containing 10% fetal bovine serum (Gibco), 1% penicillin-streptomycin, at 37°C and humidified in 5% CO_2_. Cells were passaged by trypsinization and experiments were performed from 12th to 18th passages. The detail protocol in brief is in our previous study (25).

NPC cells were from the exponential growth and cultured in 96-well plates at 5×10^3^ cells/well in 200 μl or 6-well plates at 1.5×10^5^ cells/well in 3 ml of culture medium. Each treatment condition was tested in 4 replicate wells. Either vehicle (DMSO with PBS) or rapamycin was used to treat cultured NPC cells at different concentrations (from 0 to 100 nM) at 37°C for 72 h. Then the cells in 96-well plates were counted by Cell Counting Kit-8 assay (CCK-8; Dojindo, Japan). The different numbers of cells, 5×10^3^–8×10^4^, were cultured to be counted by CCK-8 for calculation of calibration curve. Cell number was calculated using the formula: relative cell number = OD (absorbance of treated cells with medium - absorbance of only medium) × (calibration cells - constant) / OD (absorbance of calibration cells). The cells in 6-well plates were harvested for western blotting.

### In vivo studies

All procedures performed in studies involving animal experiments were approved by the Committee on the Use and Care on Animals and in accordance with the institution guidelines. Four-week-old male BALB/c nude mice were fed in the SPF grade room and anesthetized and implanted with 5×10^6^ 18th passage cultured primary NPC cells in the right flank subcutaneously. The mice were randomly separated into two groups with 6 mice in each group. Mice were intraperitoneally injected with vehicle solution (75% DMSO and 25% PBS) (26) or rapamycin (2.0 mg/kg/day) from 14th to 28th day after cell implantation. The length and width of tumors were measured every other day. The volume of the tumors was calculated as the formula: 0.4 × length × (width)^2^. At the 28th day after cell implantation, following euthanasia tumors were harvested. After weighed, parts of the tumors were fixed with 4% paraformaldehyde followed by paraffin-embedding and sectioning and parts of them were mashed and the proteins extracted. The sections were stained with hematoxylin and eosin (H&E) or immunohistochemistry. The proteins were stained with western blotting.

### Western blot analysis

NPC cells, scraped from flask or plate, and secondary tumor tissues were mashed with PBS containing 1% phenylmethyl sulfonylfluoride (PMSF) and then centrifuged to remove supernatant and sequentially proteins were isolated as the guide of protein extraction kit (Sigma, USA) with phosphorylase and metalloproteinase inhibitor cocktail. The concentrations of the proteins were assayed by BCA Protein Assay kit (Sigma). Proteins (50 μg for tissue, 20 μg for cell) were boiled at 100°C in SDS sample buffer for 5 min, electrophoresed on 12 or 8% SDS/PAGE gels, transferred to 0.45 μm bore diameter polyvinyldifluoridine membranes. Membranes were incubated overnight at 4°C stained with primary anti-human antibodies from one of the following primary antibodies: rabbit anti-mTOR, rabbit anti-P-mTOR (Ser2448), rabbit anti-4E-BP1, rabbit anti-phospho-4E-BP1 (Thr37/46), rabbit anti-MMP-2, rabbit anti-MMP-9, mouse anti-CD44 and mouse anti-SOX2 (all were 1:1,000 and from Cell Signaling Technology); rabbit anti-OCT4 (1:1,000, from Sigma). Membrane was washed with solution of PBS/0.1% Tween-20 for three times at 10 min per time, incubated at room temperature for 60 min with horseradish peroxidase-conjugated anti-rabbit or anti-mouse secondary antibody (1:5,000; Bioworld), and washed three times for 15 min with PBS/0.1% Tween-20. Peroxidase activity was visualized by chemiluminescence (NEN Life Science Products, Boston). Differences in protein expression were quantified by using a GS-710 calibrated imaging densitometer and Quantity One software (Bio-Rad).

### Immunocytochemistry and immunohistochemistry

The tissues embedded in paraffin were cut at 4-μm thickness and sections were deparaffinized with xylene twice and rehydrated with ethanol from 75 to 100%, following antigen retrieval using microwave. Cells growing on coverslips were fixed with 4% parafomaldehyde for 20 min, and then washed with PBS. After blocking peroxidase activity with 3% H_2_O_2_ (omitting for immunofluorescence), coverslips or sections were permeated by 1% Triton X-100 for 10 min and blocked by 5% goat serum in PBS for 30 min at room temperature. Primary antibodies were rabbit polyclonal anti-P-mTOR and anti-CD44 (Cell Signaling; 1:100 and 1:400), rabbit polyclonal anti-OCT4 (Sigma; 1:200). Primary antibodies were added in blocking buffer and incubated with sections at 4°C overnight. Sections were then washed with PBS and incubated with horseradish peroxidase-conjugated anti-rabbit or anti-mouse secondary antibody or goat FITC-conjugated anti-rabbit or TRITC-conjugated anti-mouse antibody (1:200) for 1 h at room temperature. A diaminobenzidine (Vector Laboratories) were used to obtain a visible reaction product. Controls for immunohistochemistry included preabsorption and coincubation of the antibodies with the corresponding antigens. Sections were dehydrated, sealed with Canada balsam or anti-fade mounting medium (Dako). A Leica microscope and a Leica digital color camera were used for examination and photography of the slides, respectively.

### Double-label immunocytochemistry

NPC tumor sections were deparaffinized, rehydrated, antigen retrieval and cultured cell coverslips were permeated, blocked as described above. The primary antibodies used were mouse anti-human CD44 (1:400) and rabbit monoclonal anti-pmTOR antigen (1:100) or mouse anti-human CD44 (1:400) and rabbit monoclonal anti-OCT4 antigen (1:200) at 4°C overnight. Then the sections or coverslips were washed with PBS three times for 15 min. The secondary antibodies were goat FITC-conjugated anti-rabbit or TRITC anti-mouse antibody (1:200) which were incubated at room temperature for 1 h. Nuclei were counterstained with DAPI. Coverslips were sealed using antifade reagent (Dako). Fluorescence signals were detected using a Leica microscope. Images were acquired using Leica digital color camera and merged using Leica digital software. Selected images were viewed at high magnification (400x). Controls will include omitting either the primary or preabsorbing primary antibody or secondary antibody.

### Statistical analysis

Results were reported as the mean ± standard deviation for each separate experiment. Data were analyzed using Student's t-test or one-way analysis of variance (ANOVA) for comparison among groups. Differences between groups were statistical significance at P<0.05.

## Results

**Figure 1:**
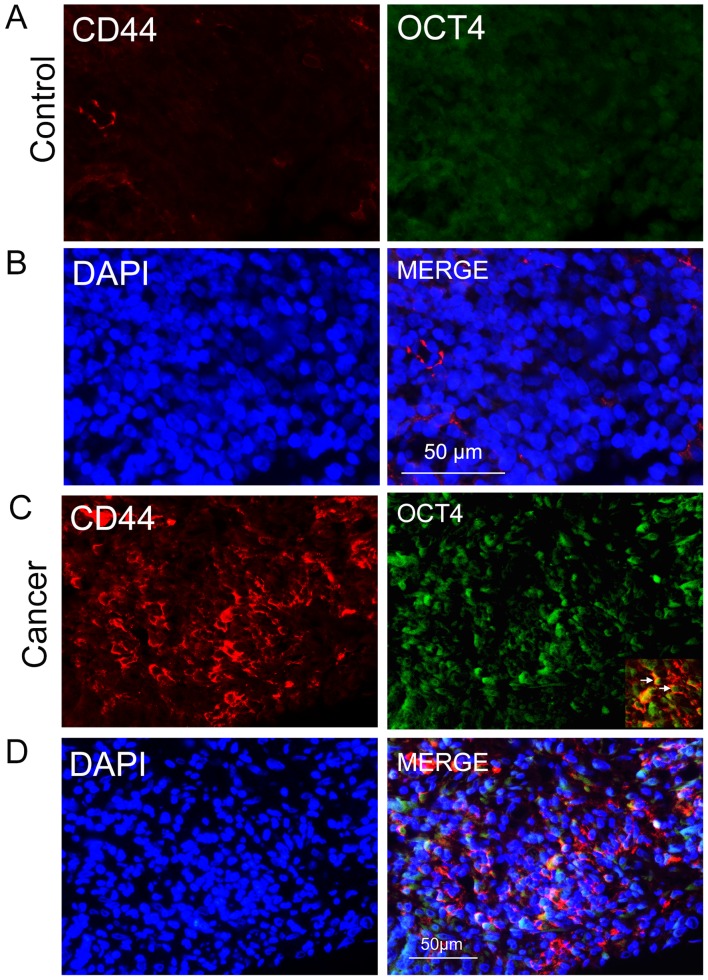
CD44-positive cancer cells in NPC tissues and nasopharyngitis epithelial cells. (A) CD44 (red) was expressed in only a few nasopharyngitis epithelial cells, and OCT4 was rarely detected in these cells. (B) The merged image shows little co-expression of CD44 and OCT4 (green) in nasopharyngitis epithelial cells. (C) CD44 and OCT4 were expressed in select regions of NPC tissue sections, mainly in epithelial areas instead of lymph regions. Double-immunolabeling showed that some regions contained co-expression of CD44 and OCT4. (D) Cell nuclei were counterstained with DAPI (blue), and the right panel shows the merged color. The magnification is 50 μm.

**Figure 2:**
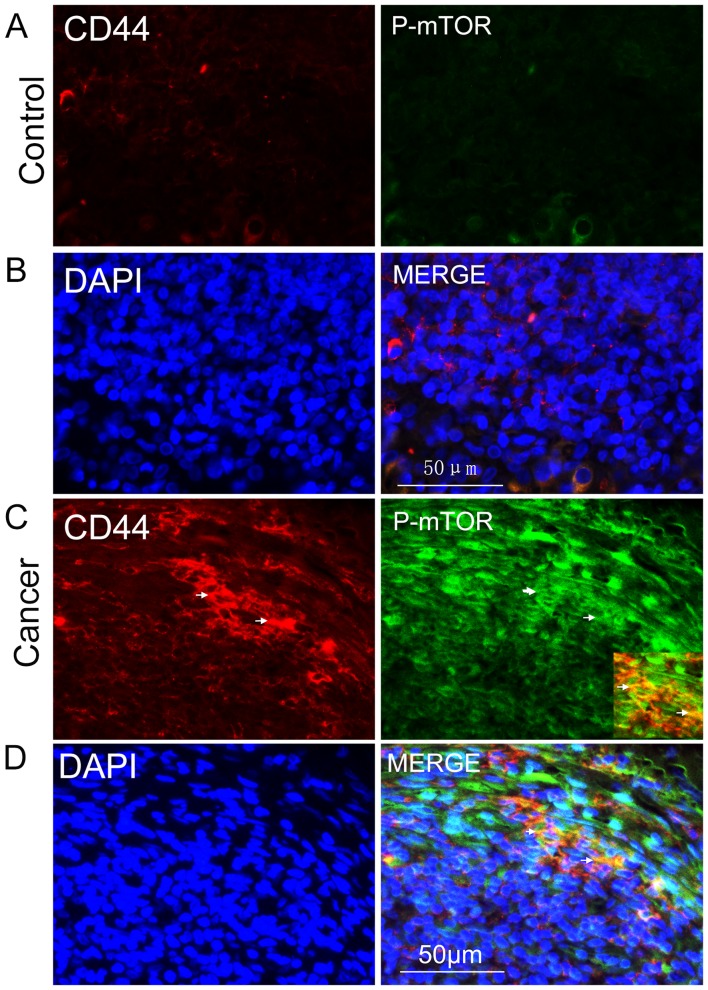
mTOR signaling pathway activation in CD44-positive cancer cells and nasopharyngitis epithelial cells. (A) P-mTOR was expressed in a very small number of nasopharyngitis epithelial cells. (B) In these cells, there was sparse co-expression of OCT4 and P-mTOR, suggesting that the mTOR signaling pathway was almost inactive. (C and D) The orange color (in the right panel) represents the region containing co-expression of CD44 (red) and P-mTOR (green). It shows that the active mTOR protein, P-mTOR, was expressed in CSC-containing regions of NPC tissue sections, suggesting that the mTOR signaling pathway was activated mainly in NPC CSCs. Cell nuclei were counterstained with DAPI (blue).

We demonstrated that mTOR signaling was activated in CD133-positive cancer cells in human primary NPC in a previous study. In the present study, our aim was to determine whether CD44 was expressed in human primary NPCs and to determine whether mTOR signaling was activated in CD44-positive cells. First, we immunohistochemically compared NPC sections with nasopharyngitis sections using an anti-CD44 antibody. In the NPC sections, we found groups of epithelial cancer cells that were CD44-positive (Fig. 1C), some of which co-expressed the stem cell biomarker OCT4 (Fig. 1C and D); the two biomarkers were rarely expressed in nasopharyngitis sections (Fig. 1A and B). This evidence suggests that the CD44/OCT4-expressing cancer cells in the NPC sections were CSCs. Many studies have reported that the mTOR signaling pathway plays a significant role in maintaining cancer cell growth dysfunction and migration; therefore, we investigated the role that mTOR signaling played in NPC CSCs. Following this, the active form of mTOR, phosphorylated mTOR, was stained for immunohistochemical detection, and we found that it was predominantly expressed in the cytoplasms of CD44-positive cells (Fig. 2C and D); it was rarely detected in controls (Fig. 2A and B). These results demonstrated the existence of CSCs in NPC and that mTOR signaling was abnormally activated in such CSCs.

**Figure 3:**
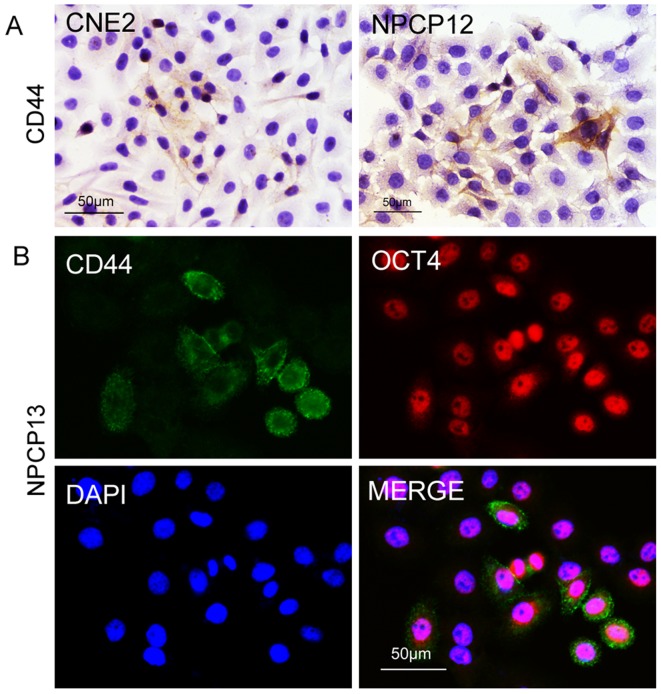
CD44, a CSC biomarker, was expressed both in the NPC cell line CNE-2 and in 12th passage cultured primary NPC cells, and it was co-expressed with another CSC biomarker, OCT4, in cultured primary NPC cells. (A) Regions of CNE-2 cells and NPC cells expressing the CSC biomarker CD44. (B) Double-immunolabeling showed that CD44-positive cells (green) were CSCs, which co-expressed OCT4 (red) in 13th passage cultured primary NPC cells. Cell nuclei were counterstained with DAPI (blue).

**Figure 4:**
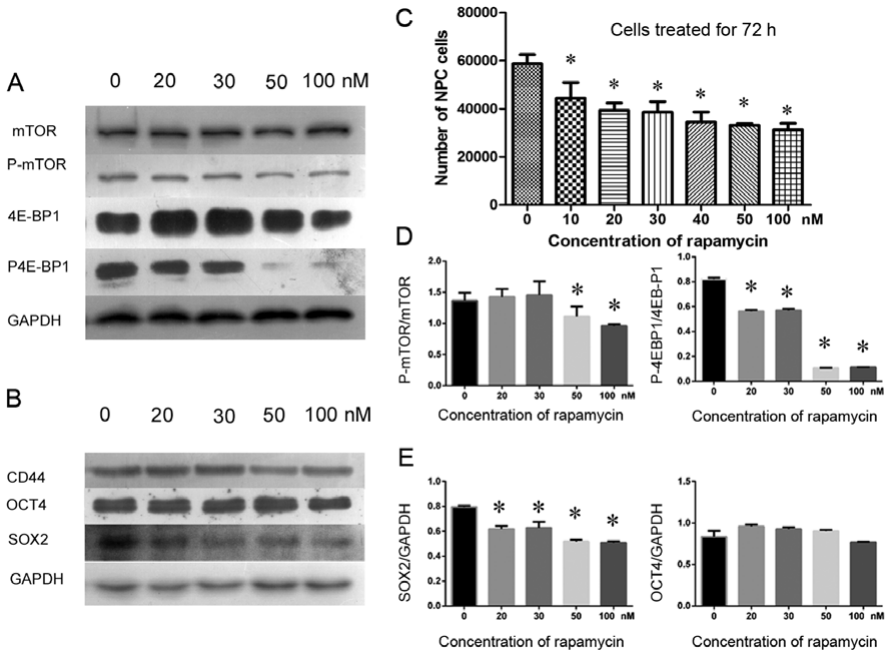
Rapamycin inhibited cell growth by inhibiting mTOR signaling in cultured primary NPC cells as detected by CCK-8 assay and western blotting. CD44 and SOX2 were suppressed by rapamycin treatment, whereas OCT4 was less affected. (A) P-mTOR and a downstream effector, the phosphorylated 4E-BP1 protein (P-4E-BP1, active 4E-BP1), were gradually suppressed as the concentration of rapamycin increased (from 20 to 100 nM). In contrast, total mTOR and 4E-BP1 were not significantly affected in cultured primary NPC cells. (B) Various concentrations of rapamycin inhibited CD44 and SOX2, but OCT4 was rarely affected. (C) The number of NPC cells decreased gradually as the dose of rapamycin (from 10 to 100 nM) increased, demonstrating that NPC cell growth was inhibited by rapamycin. (D) The relative gray value ratios of P-mTOR to mTOR and P-4E-BP1 to 4E-BP1 at various concentrations of rapamycin, in which higher concentrations of rapamycin inhibited mTOR signaling more prominently compared to the normal control group (0 nM). (E) The relative expression levels of SOX2 to GAPDH and OCT4 to GAPDH, in which SOX2 was differentially inhibited by various concentrations of rapamycin, whereas OCT4 was not suppressed in cultured primary NPC cells. ^*^P<0.05.

To decipher the important regulatory role of mTOR signaling in NPC CSCs, we performed experiments on cultured cells. To closely mimic human primary NPC, we performed the experiments using cultured primary NPC cells. We detected CD44 expression in the NPC cell line CNE2 and in 12th passage primary NPC cells. As shown in Fig. 3A, subsets of the cancer cells were CSCs. Next, we investigated whether other stem cell biomarkers were expressed in the CSCs, as the biomarkers that mark CSCs are not uniform. We found that the embryonic stem cell marker OCT4 was expressed in the CD44-positive cells, proving the expression of CD44 in NPC cells (Fig. 3B). Furthermore, the properties of NPC CSCs were studied by treating NPC cells with rapamycin to determine whether inhibition of the mTOR signaling pathway would reduce the number of CD44-positive cells and depress cancer cell proliferation. We used a CCK-8 cell count kit to assay the inhibition efficiency of rapamycin in cancer cells. The results showed that cancer cells were inhibited by rapamycin in a dose-dependent manner (Fig. 4C). Subsequently, a semiquantitative western blotting was used to investigate the expression of CD44 to determine whether the NPC CSCs were affected by rapamycin. As a result, both P-mTOR and the downstream effector, phosphorylated 4E-BP1 (P-4E-BP1), became gradually suppressed by rapamycin as the dose increased, and cell proliferation was inhibited (Fig. 4C). However, the expression levels of the mTOR and 4E-BP1 proteins were barely reduced (Fig. 4A and D). Intriguingly, rapamycin not only inhibited mTOR signaling but also depressed the expression of CD44 and SOX2 in CSCs. However, a 72-h treatment with rapamycin did not significantly reduce the expression of OCT4 (Fig. 4B and E). These data suggest that rapamycin inhibited CSCs by inhibiting mTOR signaling and that it also inhibited the proliferation of NPC cells.

**Figure 5:**
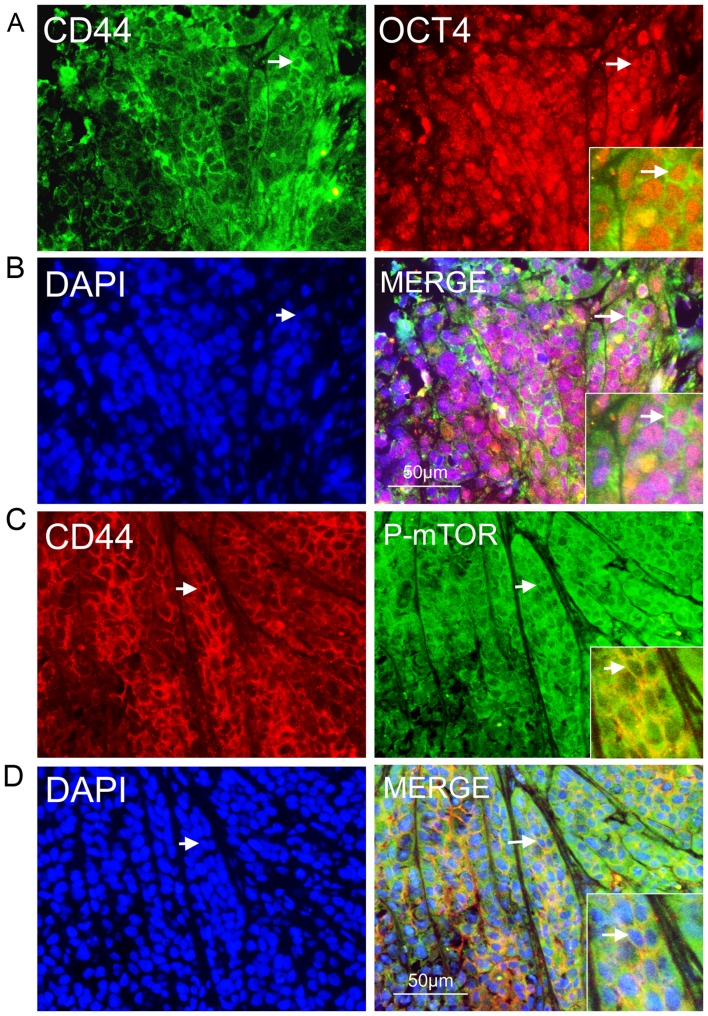
Regions of cultured primary NPC cells expressing the CSC biomarker CD44; the mTOR signaling pathway was also activated in these cells when they were injected subcutaneously into BALB/c nude mice. (A and B) CD44 and OCT4 co-expression in secondary tumor tissue sections (green, CD44; red, OCT4); (A) right panel shows co-expression in orange, while the merged color is shown in the right panel of (B-D). Double-immunostaining showed that the mTOR signaling pathway was also activated in NPC CSCs *in vivo*, in which CD44 (red) and P-mTOR (green) were co-expressed, suggesting that the mTOR signaling pathway was activated in CSCs of secondary NPCs.

**Figure 6:**
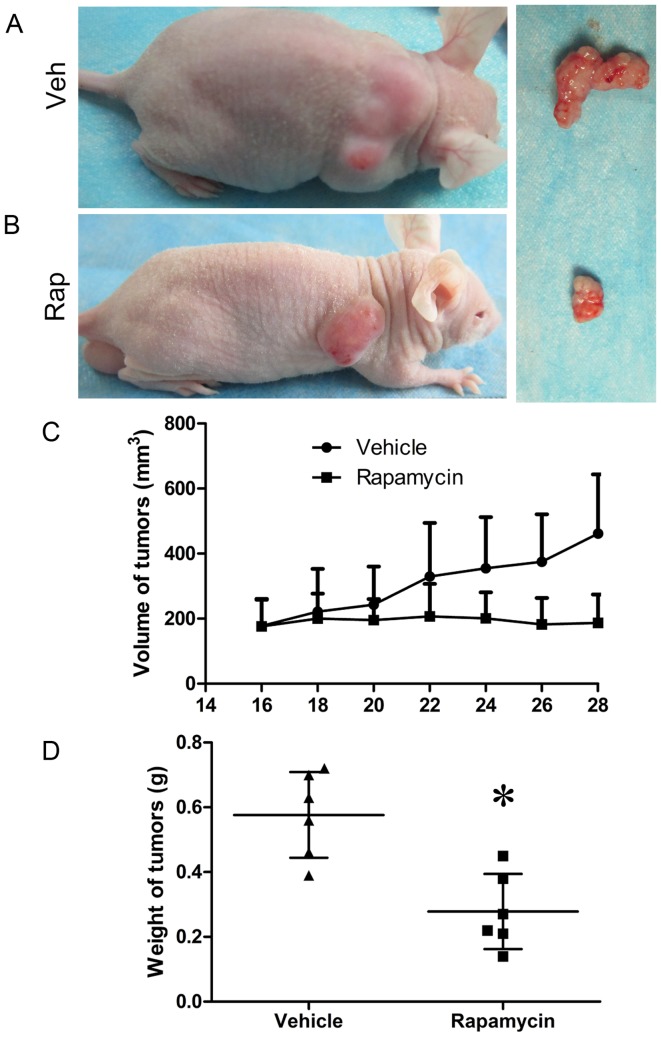
The sizes and weights of secondary tumors in BALB/c nude mice were restrained following treatment with rapamycin compared to vehicle solution. (A and B) Various sizes of secondary tumors occurring in the two groups on the same day. (C) The growth curve of secondary tumors in mice from the 2nd to the 14th day after intraperitoneal injection with rapamycin or vehicle, which showed that the tumors were inhibited by treatment with rapamycin compared to control. (D) The weights of the tumors that were harvested from the two groups, which showed that the tumors were significantly inhibited in the rapamycin group compared with the vehicle group. ^*^P<0.05. Veh, vehicle; Rap, rapamycin.

**Figure 7:**
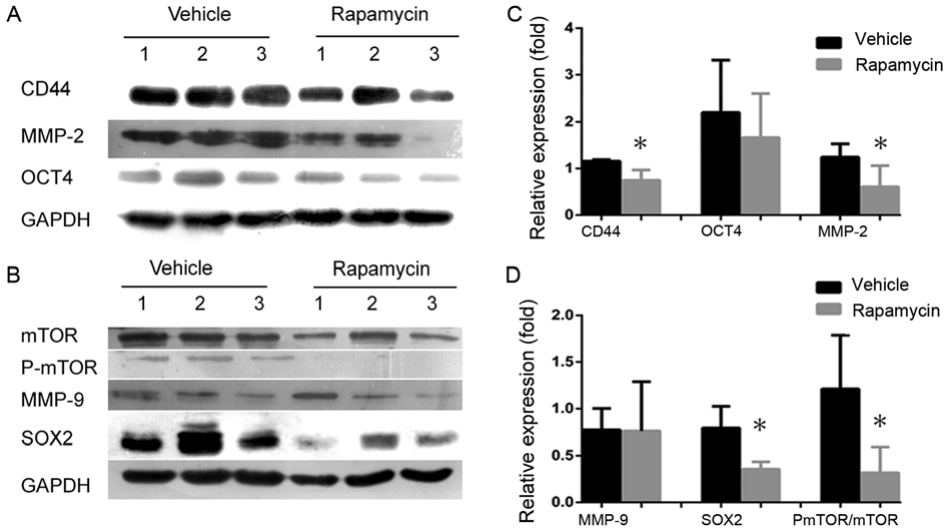
The CSC biomarkers CD44 and SOX2, as well as MMP-2, were suppressed in response to rapamycin-mediated inhibition of mTOR signaling in secondary NPCs in BALB/c nude mice, whereas OCT4 and MMP-9 were not inhibited by rapamycin. (A) CD44 and MMP-2 expression was inhibited in mice injected intraperitoneally with rapamycin compared to mice injected with control (vehicle), whereas OCT4 levels did not differ between the two groups. (B) The active form of mTOR, P-mTOR, was suppressed in NPC cells in the rapamycin group, and SOX2 was inhibited. However, MMP-9 and mTOR were not restrained by treatment with rapamycin compared to vehicle. (C and D) The relative expression levels of the above proteins in secondary tumor sections from both groups. ^*^P<0.05.

To uncover the mechanism of rapamycin-mediated inhibition of mTOR signaling in NPC cells, we investigated whether the signaling was activated in CSCs in secondary NPC tumors in BALB/c nude mice and whether rapamycin could inhibit tumor growth and NPC CSCs *in vivo*. We detected many CD44-positive NPC cells that co-expressed OCT4 *in vitro* (Fig. 5A and B); they also expressed P-mTOR (Fig. 5C and D). These results showed that mTOR signaling was activated in CSCs in secondary NPC tumors. However, it is unknown whether rapamycin affects CSC biomarker expression and tumorigenesis by inhibiting mTOR signaling *in vivo*. Therefore, we performed semiquantitative western blotting to detect CD44, SOX2 and OCT4 expression levels in secondary tumors in groups treated with either rapamycin or vehicle. CD44 and SOX2 were significantly inhibited following the inhibition of mTOR signaling (Fig. 7A and C), and the volumes and weights of tumors in rapamycin-treated mice decreased compared to those in control mice (Fig. 6). However, the expression levels of the biomarker OCT4 were not different between the two groups (Fig. 7A and C). If the properties of the CSCs were blocked by rapamycin in the secondary tumors, then the expression of migration biomarkers in cancer cells and CSCs would be reduced. Therefore, we further investigated the invasion markers MMP-2 and MMP-9 to determine whether invasion potential was suppressed following the inhibition of mTOR signaling. We found that rapamycin significantly inhibited MMP-2, but not MMP-9, compared with the control (Fig. 7B and D), suggesting that mTOR signaling plays significant roles both in maintaining NPC CSCs and in cancer progression.

## Discussion

In the present study, we investigated the expression of CD44 in NPC cells and determined whether mTOR signaling was activated in CD44-positive cells. We demonstrated that CD44 was partially expressed both in primary NPC tumor sections and in secondary tumor sections, suggesting that NPCs contain CSCs. Furthermore, we demonstrated that CD44-positive cancer cells can renew themselves or maintain their phenotypes when they are implanted in nude mice. Furthermore, these cells also expressed OCT4 and mTOR proteins, which suggested that mTOR signaling was activated in NPC CSCs. Moreover, we investigated whether rapamycin could inhibit the expression of CSC biomarkers in NPC. We found that CD44- and SOX2-positive CSCs were inhibited if we inhibited mTOR signaling, which prevents the proliferation of NPC cells and reduces secondary tumor volume and weight, but OCT4-positive CSCs were not significantly affected either *in vitro* or *in vivo*. Additionally, we detected invasion potential by evaluating MMP-2 and MMP-9 proteins, and the results showed that it was partially inhibited by rapamycin. Our findings demonstrated that mTOR signaling is activated in cancer stem cells and may play an important role in NPC tumorigenesis and progression.

CD44 is a transmembrane receptor for hyaluronan, and the binding of these two molecules plays a role in cellular behavior by activating multiple signaling pathways, as well as by affecting components involved in cell adhesion to extracellular matrix, cell proliferation, angiogenesis, tumor cell migration and cancer chemotherapy resistance (27,28). Importantly, CD44 has been identified as a biomarker of CSCs, which have the properties of self-renewal, clonal formation, and tumorigenesis *in vivo*, such as in colorectal cancer (29) and prostate cancer (30). In prostate cancer, CD44 is inhibited by rapamycin via its inhibition of mTOR signaling (31). Although CD44 has been identified as a cancer stem-like cell biomarker in NPC cell lines, its potential role in tumorigenesis has not been proven *in vivo* (17). In this experiment, we found that CD44 was expressed in select regions of NPCs and was co-expressed with the stem cell biomarker OCT4 both *in vitro* and *in vivo*, suggesting that CSCs exist in NPCs.

SOX2 and OCT4, two stem cell markers, play critical roles in maintaining self-renewal and differentiation potential in embryonic stem cells (32,33). In recent years, SOX2 and OCT4 have been identified as biomarkers of CSCs, which regulate proliferation, maintenance of self-renewal capacity, and tumorigenicity, such as in glioblastoma tumor-initiating cells (34), HNSCCs (19) and esophageal squamous cell carcinomas (35). We found that OCT4 was expressed in CD44-positive cancer cells in NPCs. We also found that SOX2, but not OCT4, is downregulated following treatment with rapamycin through the rapamycin inhibitory effect on mTOR signaling, which is accompanied by CD44 suppression. These findings suggest that rapamycin inhibits CSCs by inhibiting mTOR signaling and that various CSC biomarkers may have roles in signaling pathways.

mTOR is a highly conserved serine-threonine kinase in mammals (animals and humans). It contains two components, mTORC1 and mTORC2 (20); mTORC1 has important roles in maintaining tissue homeostasis, cell proliferation, differentiation, hematopoietic function and leukemogenesis (36,37). Increasing evidence has shown that mTOR signaling is activated in cancer cells and CSCs (38), which regulate proliferation, self-renewal and survival (39). However, the underlying mechanism of mTOR signaling in regulating the expression of CSCs is still elusive. For example, Matsumoto and colleagues found that inhibiting mTOR signaling upregulates CD133 expression in a CD133-overexpressing cancer cell line (40). Importantly, in breast cancer, rapamycin treatment inhibits the self-renewal and proliferation of breast CSCs (BCSCs), which are more susceptible to the drug than are non-BCSCs (41). Similarly, the activation of β-catenin, a component of Wnt signaling, has been associated with CD44 expression. Additionally, inhibiting the Akt pathway suppresses the expression of CD44 (42). This evidence combined with that from our study shows that signaling pathway inhibition can inhibit the expression of CSC biomarkers, suggesting a method for the therapeutic targeting of CSCs.

Matrix metalloproteinase (MMP) is a type of extracellular proteinase that binds to integrins or to CD44 to regulate the tumor microenvironment (43,44). Growing evidence shows that MMPs regulate tumor growth, basement membrane transmigration, tissue invasion, and migration (45). MMP-2 and MMP-9 have been studied often in the context of osteosarcoma (46) and pulmonary metastasis (47). An increasing number of studies have proven that MMPs are regulated by PI3K/AKT/mTOR signaling. For instance, MMP-2 is regulated by mTOR signaling and is blocked by the mTOR inhibitor rapamycin (48). Additionally, cancer metastasis is inhibited by the inhibition of MMP-9 through blocking mTOR signaling (49). However, the functions of MMP-2 and MMP-9 may be different. For example, mTOR signaling may be activated by the upregulation of MMP-9 but not by that of MMP-2 in hepatocellular carcinoma (50). Additionally, tumor volume and weight are significantly inhibited through the suppression of Akt, mTOR, and Stat3, accompanied by a decrease in the expression of MMP-2 and MMP-9 *in vivo* (51). We found that MMP-2 decreased by inhibiting CD44 via blocking mTOR signaling, whereas MMP-9 was not, demonstrating that the MMPs may have diverse roles in the signaling pathways of various cancers.

Similar to studies that have examined the role of mTOR signaling in CSCs, the regulation of CSC biomarker proteins and the process of tumorigenesis in many cancers, our results prove that the mTOR signaling pathway plays a significant role in influencing CSC behavior in human primary NPC. The effective inhibition of CSCs by rapamycin has also been confirmed. In conclusion, targeting CSCs by inhibiting mTOR signaling is an innovative therapeutic strategy for the treatment of NPC or other cancers.

## References

[1] Wei WI, Sham JS (2005). Nasopharyngeal carcinoma. Lancet.

[2] Lo KW, To KF, Huang DP (2004). Focus on nasopharyngeal carcinoma. Cancer Cell.

[3] Feng BJ, Huang W, Shugart YY, Lee MK, Zhang F, Xia JC, Wang HY, Huang TB, Jian SW, Huang P (2002). Genome-wide scan for familial nasopharyngeal carcinoma reveals evidence of linkage to chromosome 4. Nat Genet.

[4] Le QT, Tate D, Koong A, Gibbs IC, Chang SD, Adler JR, Pinto HA, Terris DJ, Fee WE, Goffinet DR (2003). Improved local control with stereotactic radiosurgical boost in patients with nasopharyngeal carcinoma. Int J Radiat Oncol Biol Phys.

[5] Shueng PW, Shen BJ, Wu LJ, Liao LJ, Hsiao CH, Lin YC, Cheng PW, Lo WC, Jen YM, Hsieh CH (2011). Concurrent image-guided intensity modulated radiotherapy and chemotherapy following neoadjuvant chemotherapy for locally advanced nasopharyngeal carcinoma. Radiat Oncol.

[6] Wang J, Guo LP, Chen LZ, Zeng YX, Lu SH (2007). Identification of cancer stem cell-like side population cells in human nasopharyngeal carcinoma cell line. Cancer Res.

[7] Yang YP, Chien Y, Chiou GY, Cherng JY, Wang ML, Lo WL, Chang YL, Huang PI, Chen YW, Shih YH (2012). Inhibition of cancer stem cell-like properties and reduced chemoradioresistance of glioblastoma using microRNA145 with cationic polyurethane-short branch PEI. Biomaterials.

[8] Bao S, Wu Q, McLendon RE, Hao Y, Shi Q, Hjelmeland AB, Dewhirst MW, Bigner DD, Rich JN (2006). Glioma stem cells promote radioresistance by preferential activation of the DNA damage response. Nature.

[9] Bonnet D, Dick JE (1997). Human acute myeloid leukemia is organized as a hierarchy that originates from a primitive hematopoietic cell. Nat Med.

[10] Singh SK, Hawkins C, Clarke ID, Squire JA, Bayani J, Hide T, Henkelman RM, Cusimano MD, Dirks PB (2004). Identification of human brain tumour initiating cells. Nature.

[11] Ginestier C, Hur MH, Charafe-Jauffret E, Monville F, Dutcher J, Brown M, Jacquemier J, Viens P, Kleer CG, Liu S (2007). ALDH1 is a marker of normal and malignant human mammary stem cells and a predictor of poor clinical outcome. Cell Stem Cell.

[12] Krivtsov AV, Twomey D, Feng Z, Stubbs MC, Wang Y, Faber J, Levine JE, Wang J, Hahn WC, Gilliland DG (2006). Transformation from committed progenitor to leukaemia stem cell initiated by MLL-AF9. Nature.

[13] Mani SA, Guo W, Liao MJ, Eaton EN, Ayyanan A, Zhou AY, Brooks M, Reinhard F, Zhang CC, Shipitsin M (2008). The epithelial-mesenchymal transition generates cells with properties of stem cells. Cell.

[14] Morel AP, Lièvre M, Thomas C, Hinkal G, Ansieau S, Puisieux A (2008). Generation of breast cancer stem cells through epithelial-mesenchymal transition. PLoS One.

[15] Suvà ML, Riggi N, Stehle JC, Baumer K, Tercier S, Joseph JM, Suvà D, Clément V, Provero P, Cironi L (2009). Identification of cancer stem cells in Ewing's sarcoma. Cancer Res.

[16] Prince ME, Sivanandan R, Kaczorowski A, Wolf GT, Kaplan MJ, Dalerba P, Weissman IL, Clarke MF, Ailles LE (2007). Identification of a subpopulation of cells with cancer stem cell properties in head and neck squamous cell carcinoma. Proc Natl Acad Sci USA.

[17] Su J, Xu XH, Huang Q, Lu MQ, Li DJ, Xue F, Yi F, Ren JH, Wu YP (2011). Identification of cancer stem-like CD44^+^ cells in human nasopharyngeal carcinoma cell line. Arch Med Res.

[18] Bareiss PM, Paczulla A, Wang H, Schairer R, Wiehr S, Kohlhofer U, Rothfuss OC, Fischer A, Perner S, Staebler A (2013). SOX2 expression associates with stem cell state in human ovarian carcinoma. Cancer Res.

[19] Koo BS, Lee SH, Kim JM, Huang S, Kim SH, Rho YS, Bae WJ, Kang HJ, Kim YS, Moon JH (2015). Oct4 is a critical regulator of stemness in head and neck squamous carcinoma cells. Oncogene.

[20] Engelman JA (2009). Targeting PI3K signalling in cancer: Opportunities, challenges and limitations. Nat Rev Cancer.

[21] Navé BT, Ouwens M, Withers DJ, Alessi DR, Shepherd PR (1999). Mammalian target of rapamycin is a direct target for protein kinase B: Identification of a convergence point for opposing effects of insulin and amino-acid deficiency on protein translation. Biochem J.

[22] Kolev VN, Wright QG, Vidal CM, Ring JE, Shapiro IM, Ricono J, Weaver DT, Padval MV, Pachter JA, Xu Q (2015). PI3K/ mTOR dual inhibitor VS-5584 preferentially targets cancer stem cells. Cancer Res.

[23] Cao Y, Liu X, Lu W, Chen Y, Wu X, Li M, Wang XA, Zhang F, Jiang L, Zhang Y (2015). Fibronectin promotes cell proliferation and invasion through mTOR signaling pathway activation in gallbladder cancer. Cancer Lett.

[24] Shanmugarantnam KSL (1991). Histological Typing of Tumors of the Upper Respiratory Tract and Ear.

[25] Yang C, Peng J, Jiang W, Zhang Y, Chen X, Wu X, Zhu Y, Zhang H, Chen J, Wang J (2013). mTOR activation in immature cells of primary nasopharyngeal carcinoma and anti-tumor effect of rapamycin in vitro and in vivo. Cancer Lett.

[26] Bhola P, Banerjee S, Mukherjee J, Balasubramanium A, Arun V, Karim Z, Burrell K, Croul S, Gutmann DH, Guha A (2010). Preclinical in vivo evaluation of rapamycin in human malignant peripheral nerve sheath explant xenograft. Int J Cancer.

[27] Turley EA, Noble PW, Bourguignon LY (2002). Signaling properties of hyaluronan receptors. J Biol Chem.

[28] Wang SJ, Bourguignon LY (2006). Hyaluronan and the interaction between CD44 and epidermal growth factor receptor in oncogenic signaling and chemotherapy resistance in head and neck cancer. Arch Otolaryngol Head Neck Surg.

[29] Du L, Wang H, He L, Zhang J, Ni B, Wang X, Jin H, Cahuzac N, Mehrpour M, Lu Y (2008). CD44 is of functional importance for colorectal cancer stem cells. Clin Cancer Res.

[30] Patrawala L, Calhoun T, Schneider-Broussard R, Li H, Bhatia B, Tang S, Reilly JG, Chandra D, Zhou J, Claypool K (2006). Highly purified CD44^+^ prostate cancer cells from xenograft human tumors are enriched in tumorigenic and metastatic progenitor cells. Oncogene.

[31] Wang J, Lu Y, Wang J, Koch AE, Zhang J, Taichman RS (2008). CXCR6 induces prostate cancer progression by the AKT/ mammalian target of rapamycin signaling pathway. Cancer Res.

[32] Fong YW, Inouye C, Yamaguchi T, Cattoglio C, Grubisic I, Tjian R (2011). A DNA repair complex functions as an Oct4/Sox2 coactivator in embryonic stem cells. Cell.

[33] Zhou HY, Katsman Y, Dhaliwal NK, Davidson S, Macpherson NN, Sakthidevi M, Collura F, Mitchell JA (2014). A Sox2 distal enhancer cluster regulates embryonic stem cell differentiation potential. Genes Dev.

[34] Gangemi RM, Griffero F, Marubbi D, Perera M, Capra MC, Malatesta P, Ravetti GL, Zona GL, Daga A, Corte G (2009). SOX2 silencing in glioblastoma tumor-initiating cells causes stop of proliferation and loss of tumorigenicity. Stem Cells.

[35] Shahryari A, Rafiee MR, Fouani Y, Oliae NA, Samaei nM, Shafiee M, Semnani S, Vasei M, Mowla SJ (2014). Two novel splice variants of SOX2OT, SOX2OT-S1, and SOX2OT-S2 are coupregulated with SOX2 and OCT4 in esophageal squamous cell carcinoma. Stem Cells.

[36] Yuan TL, Cantley LC (2008). PI3K pathway alterations in cancer: Variations on a theme. Oncogene.

[37] Kalaitzidis D, Sykes SM, Wang Z, Punt N, Tang Y, Ragu C, Sinha AU, Lane SW, Souza AL, Clish CB (2012). mTOR complex 1 plays critical roles in hematopoiesis and Pten-loss-evoked leukemogenesis. Cell Stem Cell.

[38] Gaur P, Sceusi EL, Samuel S, Xia L, Fan F, Zhou Y, Lu J, Tozzi F, Lopez-Berestein G, Vivas-Mejia P (2011). Identification of cancer stem cells in human gastrointestinal carcinoid and neuroendocrine tumors. Gastroenterology.

[39] Gulhati P, Cai Q, Li J, Liu J, Rychahou PG, Qiu S, Lee EY, Silva SR, Bowen KA, Gao T (2009). Targeted inhibition of mammalian target of rapamycin signaling inhibits tumorigenesis of colorectal cancer. Clin Cancer Res.

[40] Matsumoto K, Arao T, Tanaka K, Kaneda H, Kudo K, Fujita Y, Tamura D, Aomatsu K, Tamura T, Yamada Y (2009). mTOR signal and hypoxia-inducible factor-1 alpha regulate CD133 expression in cancer cells. Cancer Res.

[41] Chang WW, Lin RJ, Yu J, Chang WY, Fu CH, Lai A, Yu JC, Yu AL (2013). The expression and significance of insulin-like growth factor-1 receptor and its pathway on breast cancer stemprogenitors. Breast Cancer Res.

[42] Li J, Zhou BP (2011). Activation of β-catenin and Akt pathways by Twist are critical for the maintenance of EMT associated cancer stem cell-like characters. BMC Cancer.

[43] Egeblad M, Werb Z (2002). New functions for the matrix metalloproteinases in cancer progression. Nat Rev Cancer.

[44] Kessenbrock K, Plaks V, Werb Z (2010). Matrix metalloproteinases: Regulators of the tumor microenvironment. Cell.

[45] Hotary K, Li XY, Allen E, Stevens SL, Weiss SJ (2006). A cancer cell metalloprotease triad regulates the basement membrane transmigration program. Genes Dev.

[46] Bjørnland K, Flatmark K, Pettersen S, Aaasen AO, Fodstad O, Maelandsmo GM (2005). Matrix metalloproteinases participate in osteosarcoma invasion. J Surg Res.

[47] van Kempen LC, Coussens LM (2002). MMP9 potentiates pulmonary metastasis formation. Cancer Cell.

[48] Zhang D, Bar-Eli M, Meloche S, Brodt P (2004). Dual regulation of MMP-2 expression by the type 1 insulin-like growth factor receptor: The phosphatidylinositol 3-kinase/Akt and Raf/ ERK pathways transmit opposing signals. J Biol Chem.

[49] Liu SC, Chen C, Chung CH, Wang PC, Wu NL, Cheng JK, Lai YW, Sun HL, Peng CY, Tang CH (2014). Inhibitory effects of butein on cancer metastasis and bioenergetic modulation. J Agric Food Chem.

[50] Chen JS, Wang Q, Fu XH, Huang XH, Chen XL, Cao LQ, Chen LZ, Tan HX, Li W, Bi J (2009). Involvement of PI3K/PTEN/ AKT/mTOR pathway in invasion and metastasis in hepatocellular carcinoma: Association with MMP-9. Hepatol Res.

[51] Lv C, Kong H, Dong G, Liu L, Tong K, Sun H, Chen B, Zhang C, Zhou M (2014). Antitumor efficacy of α-solanine against pancreatic cancer in vitro and in vivo. PLoS One.

